# Magic Silicon Dioxide for Widely Tunable Photonic Integrated Circuits

**DOI:** 10.1021/acsphotonics.4c01373

**Published:** 2025-02-05

**Authors:** Bruno Lopez-Rodriguez, Naresh Sharma, Zizheng Li, Roald van der Kolk, Jasper van der Boom, Thomas Scholte, Jin Chang, Simon Gröblacher, Iman Esmaeil Zadeh

**Affiliations:** † Department of Imaging Physics (ImPhys), Faculty of Applied Sciences, Delft University of Technology, 2628 CJ Delft, The Netherlands; ‡ Department of Quantum Nanoscience, Faculty of Applied Sciences, Delft University of Technology, 2628 CJ Delft, The Netherlands

**Keywords:** silicon dioxide, strain, thermo-optic tunability, athermal, integrated photonics

## Abstract

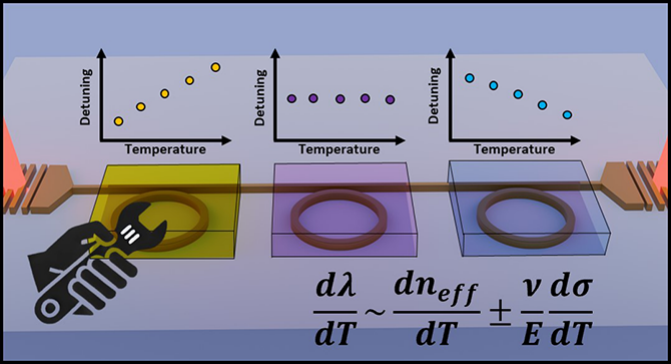

Integrated photonic circuits have transformed data communication, biosensing, and light detection and ranging and hold wide-ranging potential for optical computing, optical imaging, and signal processing. These applications often require tunable and reconfigurable photonic components, most commonly accomplished through the thermo-optic effect. However, the resulting tuning window is limited for standard optical materials, such as silicon dioxide and silicon nitride. Most importantly, bidirectional thermal tuning on a single platform has not been realized. For the first time, we show that by tuning and optimizing the deposition conditions in inductively coupled plasma chemical vapor deposition (ICPCVD) of silicon dioxide, this material can be used to deterministically tune the thermo-optic properties of optical devices without introducing significant losses. We demonstrate that we can deterministically integrate positive and negative wavelength shifts on a single chip, validated on amorphous silicon carbide (a-SiC), silicon nitride (SiN), and silicon-on-insulator (SOI) platforms. This enables the fabrication of a novel tunable coupled ring optical waveguide (CROW) requiring only a single heater. In addition, we observe up to a 10-fold improvement of the thermo-optic tunability and demonstrate athermal ring resonators with shifts as low as 1.5 pm/°C. The low-temperature deposition of our silicon dioxide cladding can be combined with lift-off to isolate the optical devices, resulting in a decrease in thermal crosstalk by at least 2 orders of magnitude. Our method paves the way for novel photonic architectures incorporating bidirectional thermo-optic tunability.

## Introduction

Achieving a high degree of tunability in photonic devices has been a focal point in the field of integrated photonics for several decades with a wide range of applications from telecommunications and biochemical sensing to fundamental quantum photonic experiments in many material platforms.^1−17^

The most universally utilized method to achieve photonic device tunability is by exploiting the thermo-optic effect. The thermo-optic coefficient (TOC) of an optical material describes the change in refractive index due to a temperature change (d*n*/d*T*).^18−20^ It has been shown that the thermal tunability of a platform depends on the volume expansion of the materials, the temperature-induced refractive index differences, waveguide path-difference variations, the strain between core and cladding material, and their mechanical properties such as Young modulus and Poisson constant, described with the following relation and simplified^21−24^
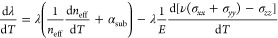
1where *n*_eff_ is the effective refractive index, α_sub_ is the thermal expansion coefficient of the substrate, *E* and ν are the Young modulus and the Poisson constant of the core, respectively, and σ_*xx*_, σ_*yy*_, and σ_*zz*_ are the stress components. In eq 1 the first term on the right-hand side represents the effective thermo-optical coefficient and is the usual term used in literature studies for the stress-free state. In contrast, the second term corresponds to the thermal shift produced by a stress gradient. Thermal stress arises due to the mismatch between the thermal expansion coefficients of the waveguide and the cladding materials. In the usual configuration to exploit the thermo-optic effect, metal heaters are placed above the guiding material to control the phase of the light. This method of tuning photonic devices is virtually lossless, easy to integrate, and applicable to nearly all photonic platforms. Nevertheless, the tuning strength is specific to the material platform and, importantly, it is weak in photonic platforms such as silicon nitride or silicon dioxide, two of the most commonly used materials in integrated photonics.^25,26^ To compensate for it, hybrid integration with platforms with higher TOC can be performed such that, e.g.: delay lines can be fabricated on a low-loss material (SiN) while interference is done on high TOC platforms (a-SiC).^27−29^ Increasing the TOC of materials has been a major challenge and, so far, accomplished by tuning their composition^30,31^ or depositing high refractive index claddings such as silicon oxycarbide (SiOC).^32^ Using TiO_2_, a high index cladding, several works achieved silicon-on-insulator (SOI) athermal devices, where this material cancels out the positive thermal expansion of this photonic platform.^33−36^ However, these methods are complex and are applicable only to specific platforms. Moreover, changing the composition modifies the overall properties of the guiding layer and often significantly increases the propagation losses, while depositing a high index cladding increases the bending losses and reduces the integration density. Other works have shown that the thermo-optic properties of optical devices can also be modified by applying external thermal stress, with the drawback of presenting multimode operation, birefringence, and loss increase.^21,22^ Finally, only positive or negative thermal shifts have been achieved thus far;^37−39^ bidirectional tuning on a single platform remains elusive.

**Figure 1:**
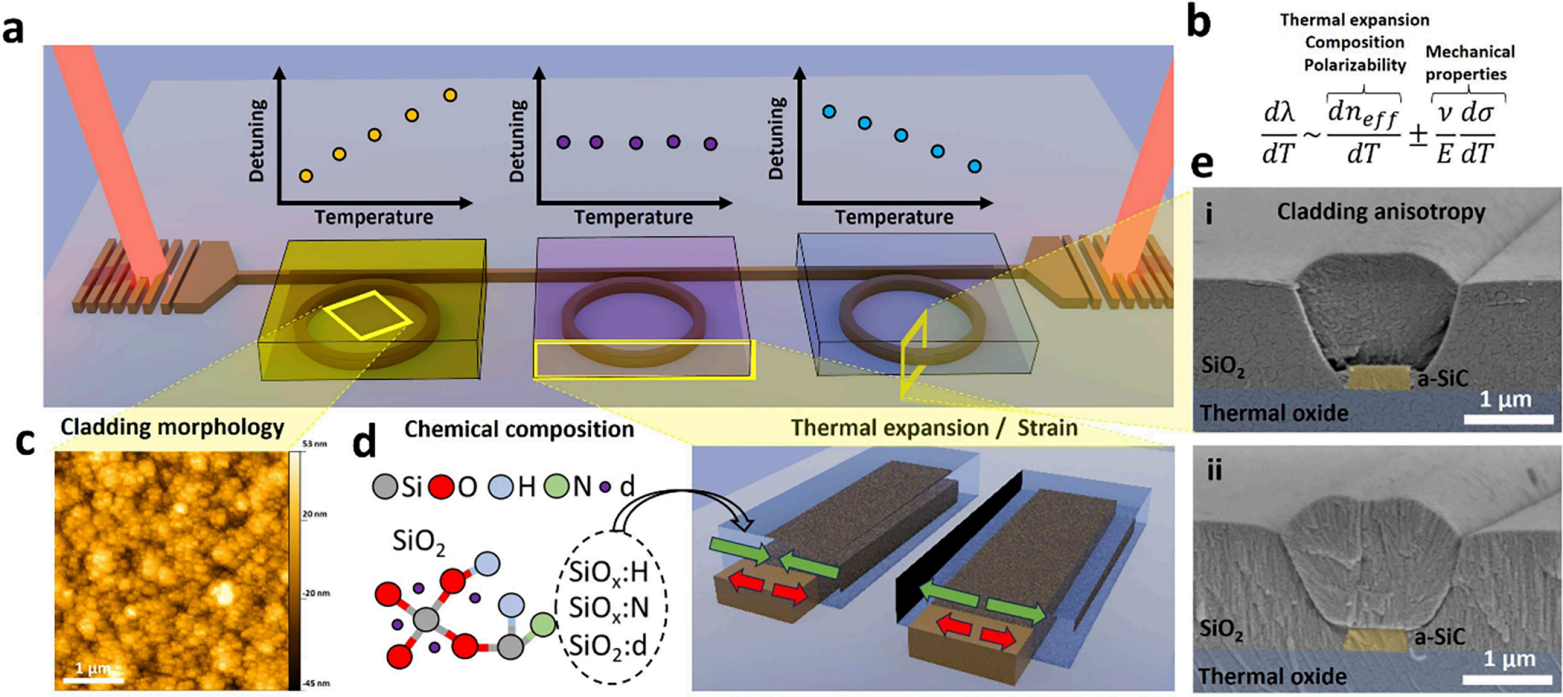
(a) Illustration of the deterministic integration of different claddings on the same chip with positive, athermal, and negative detuning. (b) General formula for the thermal shift in optical devices, proportional to volume expansion, composition, polarizability, and mechanical properties.^24,40^ Varying the deposition parameters in CVD techniques can induce changes in the thermal expansion properties of the films due to (c) the morphology (grain size and topography)^45^ and density^46^ of the films as shown by our AFM studies, (d) their chemical composition and bonds with other compounds such as hydrogen, nitrogen, or dopants,^47,48^ and (e) cladding anisotropy around the waveguide adding additional strain,^23,49^ as has also been observed in our cross-ectional SEM images of waveguides with ICPCVD silicon dioxide claddings deposited at chamber pressures of (i) 2.5 mTorr and (ii) 12 mTorr.

In this work, we report for the first time that inductively coupled plasma chemical vapor deposition (ICPECVD) can be used to tailor the thermo-optic properties of optical devices by depositing silicon dioxide claddings, the most common optical material, achieving large positive and negative thermal wavelength shifts on a single chip without significantly affecting the optical losses (depicted in Figure 1a). We apply this technique on amorphous silicon carbide, silicon nitride and silicon-on-insulator platforms and demonstrate an up to 10-fold improvement of the thermo-optical wavelength tunability of SiN compared to literature values. Moreover, we demonstrate a 5-fold higher thermal tunability and athermal photonic ring resonators on an a-SiC platform. This powerful tunability range allows us to showcase unprecedented photonic devices by deterministically including claddings with negative and positive thermal responses on the same chip. Additionally, thanks to our low-temperature deposition technique, we introduce a novel fabrication approach to isolate active optical devices and demonstrate a decrease in thermal crosstalk by at least 2 orders of magnitude.

## Results and Discussion

### Thermo-Optic Wavelength Shift and Propagation Loss

**Figure 2:**
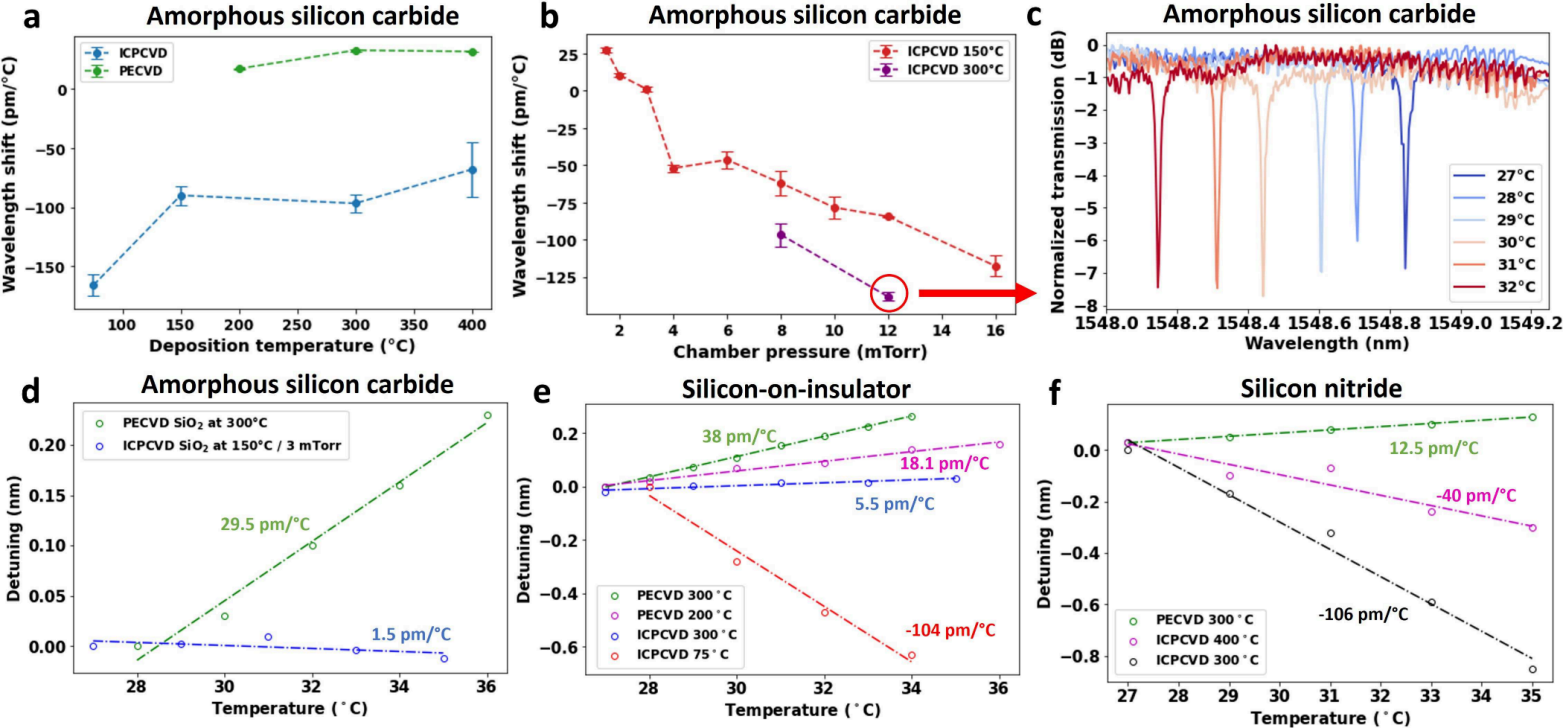
(a) Wavelength shift in pm/°C for a-SiC devices with ICPCVD cladding deposited at different temperatures and a constant chamber pressure of 8 mTorr using ICPCVD (green) and PECVD (blue) together with the standard deviation obtained from the linear fitting. Lines are guides for the eye. (b) Thermo-optic tunability in pm/°C for a-SiC devices with ICPCVD cladding deposited at different chamber pressures and temperatures of 150 °C (red) and 300 °C (purple) together with the standard deviation obtained from the linear fitting. (c) Wavelength spectra of a device with a cladding deposited via ICPCVD at 300 °C and 12 mTorr chamber pressure and recorded at different temperatures between 27 and 32 °C. (d) Detuning of the resonance wavelength for a-SiC devices with cladding deposited by PECVD (chamber temperature 300 °C; green symbols) and ICPCVD (chamber temperature 150 °C and chamber pressure 3 mTorr; blue symbols). Lines are linear fits. (e) Detuning of the resonance wavelength for silicon-on-insulator optical devices with PECVD and ICPCVD claddings deposited at different temperatures. Lines are linear fits with slopes, as indicated. (f) Detuning of the resonance wavelength for silicon nitride optical devices with PECVD and ICPCVD claddings deposited at different temperatures. Lines are linear fits with slopes as indicated.

Following the general formula for the thermo-optic coefficient in Figure 1b and, as demonstrated in other works, applying thermal stress in the cladding can contribute to controlling the temperature sensitivity of optical devices.^21−24^ In CVD techniques it is known that different parameters such as temperature, chamber pressure, gas ratios, and RF plasma power can modify the stress profiles in the deposited films,^40−44^ their morphology (Figure 1c) such as grain size^45^ and density^46^ or the chemical composition (Figure 1d), with nitrogen, hydrogen and incorporation of dopants, overall affecting the thermal expansion properties of the cladding.^47,48^ Most importantly it has been shown that, in ICPCVD, RF plasma power and pressure can also induce noticeable differences in the anisotropy ratio, which relates the film thickness in the sidewall to that of the substrate and could potentially affect the strain profile around the waveguide region.^49^Figure 1e shows two scanning electron microscope cross sections of the deposition of silicon dioxide around the waveguide region. Different chamber pressures of (i) 2.5 and (ii) 12 mTorr produce noticeable differences in the anisotropy of the cladding. These parameters can also affect other contributing factors such as the thermal expansion coefficient and polarizability.^24^ We measure the thermal response of optical ring resonators (radius 120 μm, waveguide width 750 nm, gap 850 nm and, as measured by ellipsometry, thickness of 270 nm) fabricated on a-SiC films and covered with silicon dioxide claddings deposited via ICPCVD and PECVD techniques under different deposition temperatures (Figure 2a) and chamber pressures (Figure 2b). Details about free spectral range, group index, effective index, thermal tunability, and device dimensions for a-SiC, SiN, and Si platforms can be found in the Supporting Information together with the calculated effective TOC. For compatibility with the lift-off process, we tune the thermal tunability at a fixed temperature of 150 °C by modifying the chamber pressure. We conducted temperature reliability tests for different claddings and found that silicon dioxide claddings deposited at 150 °C can withstand temperatures up to 400 °C (Supporting Information). At a deposition temperature of 150 °C (Figure 2b) we achieve wavelength shifts between +29.5 pm/°C (at 2 mTorr) and −118 pm/°C (at 16 mTorr). Using this approach, we record a thermal shift of −138 pm/°C in the a-SiC platform depositing ICPCVD SiO_2_ at 300 °C and 12 mTorr, corresponding to d*n*_eff_/d*T* = −2.2 × 10^–4^. The respective spectra with detuning of the resonance dip at different temperatures can be seen in Figure 2c. This represents a tunability almost 5 times higher than those of standard devices^27^ and significantly 22% more than that of silicon^50^ (see the Supporting Information for the fitting). Crucially, for sensing applications, and thanks to the significant thermal tuning from negative to positive, our method allows for the fabrication of athermal devices by choosing the appropriate chamber pressure (3 mTorr) and deposition temperature (150 °C). We achieved a thermal response as low as 1.5 pm/°C in a temperature range between 27 and 35 °C, a relevant temperature range for biological and chemical sensing^51^ (Figure 2d), which is 20 times lower than for the standard PECVD-cladded devices (see the Supporting Information for individual spectra). In Figure 2a it can be seen that the largest thermal tunability of −166 pm/°C occurs for a deposition temperature of 75 °C (temperature increases to 91 °C due to table heating). The spectra and fits can be found in the Supporting Information. For these low temperatures, we find that the device response is not stable, resulting in different TOCs after the temperature is raised. As shown in the Supporting Information, the same is true for a device made on a silicon nitride platform, a cladding deposited at 30 °C cannot be heated more than 33 °C since the thermal shift and hence the tunability decreases.

To demonstrate that this method can be applied to other platforms, we deposit claddings using PECVD and ICPCVD at different temperatures on SOI (width 700 nm and thickness 220 nm with ring radius of 120 μm) and SiN (width 1000 nm and thickness 368 nm with ring radius of 120 μm) platforms and record the detuning of the resonance wavelength for each device as a function of the stage temperature. The resonance wavelength detuning as a function of temperature is shown in Figure 2e,f, respectively. The representative spectra at different temperatures can be found in the Supporting Information. Figure 2e shows that for SOI optical ring resonators we can achieve thermal shifts between −96 pm/°C (d*n*_eff_/d*T* = −2.2 × 10^–4^/°C) for ICPCVD oxide deposited at 75 °C and +40 pm/°C for 300 °C PECVD oxide cladding.

Similarly, Figure 2f shows that depositing PECVD SiO_2_ on SiN devices yields 14 pm/°C, comparable to values found in the literature.^52^ In contrast, when this cladding is deposited with ICPCVD at 300 °C we achieve a thermal shift of −106 pm/°C, representing an improvement of almost an order of magnitude and a d*n*_eff_/d*T* value of −1.2 × 10^–4^/°C.

In terms of optical quality, using ICPCVD SiO_2_ at a temperature of 150 °C and changing the chamber pressure results in devices with similar quality over the pressure range from 2 to 8 mTorr with waveguide propagation losses of 2.68 dB/cm (*Q*_int_ = 1.58 × 10^5^, comparable to literature values).

### Passive and Active Devices

**Figure 3:**
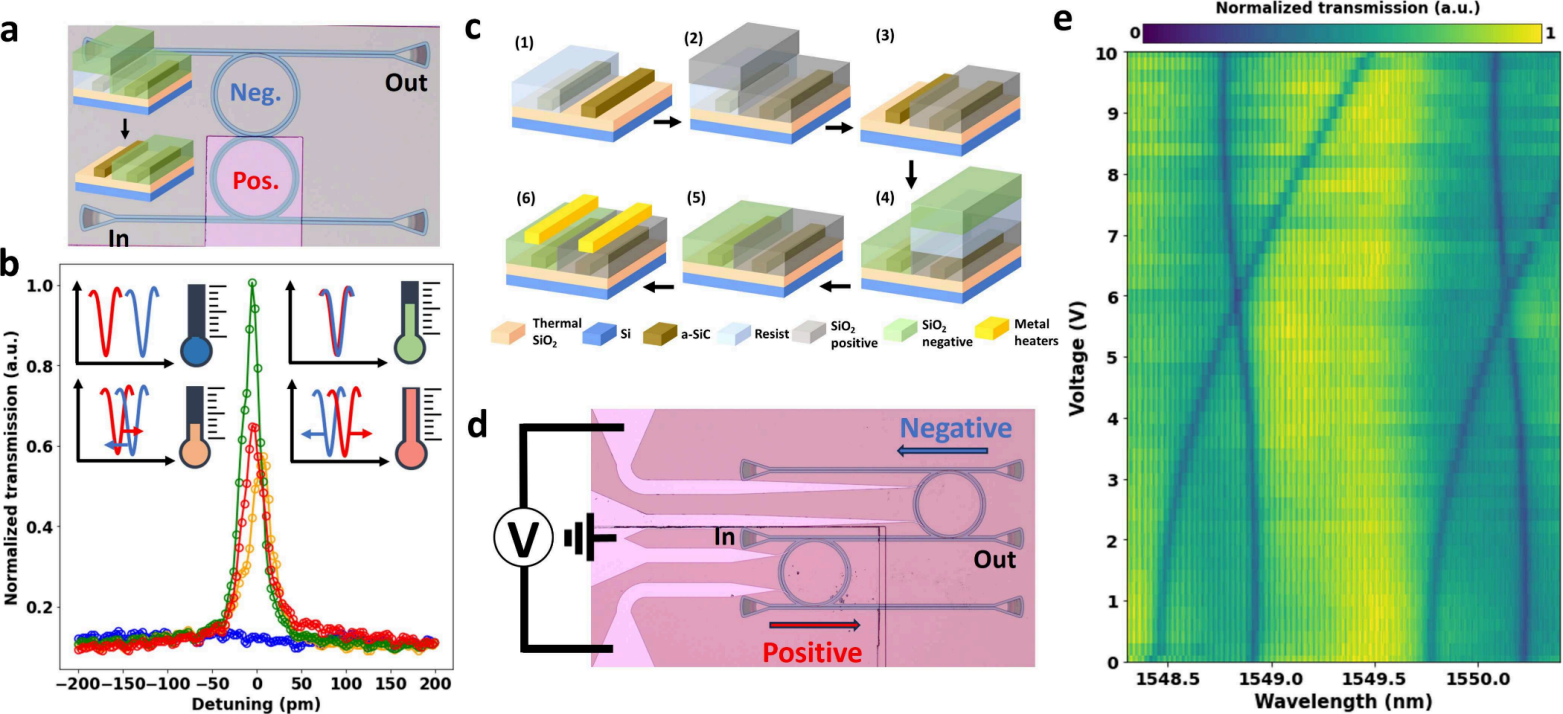
(a) CROW resonator fabricated using a bidirectional thermal response with only one cladding. Inset: basic lift-off process with cladding. (b) Transmission spectra around 1552 nm of the output signal of the device shown in (d) at temperatures of 32.5 °C (blue), 33.5 °C (orange), 33.9 °C (green), and 34.5 °C (red). (c) Fabrication scheme for the inclusion of bidirectional claddings in optical devices: (1) resist spin coating, exposure, and development; (2) SiO_2_ cladding deposition for positive shift; (3) lift-off in acetone; (4) resist spin-coating, exposure, development, and deposition of negative TOC cladding; (5) lift-off in acetone; (6) patterning of metal heaters via lift-off. (d) Optical microscope image of two ring resonators connected with a middle waveguide. (e) Resonant wavelength as a function of the voltage applied to metal heaters of the optical device shown in (b (*R* = 784 Ω).

To highlight the flexibility of our fabrication strategy, we propose the first demonstration of a passive actuated coupled resonator optical waveguide (CROW) device using a single cladding with negative thermal tunability. CROW devices are typically used in optical filtering, dispersion compensation, and nonlinear optics.^53−56^ In addition, they can be used to delay, store, and buffer photons with controlled times.^57^ The basic structure of a CROW device consists of two or more adjacent and coupled ring resonators. Figure 3a shows a device with this configuration and the inset shows a basic lift-off process using a silicon dioxide cladding deposited at 150 °C and 8 mTorr. Lift-off is challenging for CVD methods due to the high deposition temperatures, incompatible with lithography resists.^58^ Other techniques such as sputtering can be used, but the number of parameters that can be modified is much more limited and they often lead to lower-quality oxides (unless annealing is performed) and poor step coverage.^59,60^Figure 3b shows the output spectra as the temperature of the sample is increased from 32.5 to 34.5 °C. In this case, the matching condition is achieved at a temperature of 33.9 °C. As another example, we show in the Supporting Information a Mach–Zehnder interferometer where it is possible to vary the output power in the ports by modifying the stage temperature. In their usual configuration, these devices are tuned with separate microheaters on top of the rings. Once the coupling condition is fulfilled, the resonances of each ring overlap and the photons can be filtered to the output. In contrast, our CROW design, incorporating two rings of opposite thermal shifts, can be tuned by a single heater. This is done by using a lift-off friendly temperature of 150 °C, compatible with lithography resists, but with chamber pressures of 2 mTorr (positive TOC) and 8 mTorr (negative TOC) following Figure 2b.

Using our fabrication scheme, we fabricate a CROW configuration with two claddings (Figure 3c) and ring parameters similar to those previously shown. Figure 3d shows an optical microscope image of the final rings with heaters. They are connected in series (or in parallel) having a common voltage and common ground. For a device connected in parallel (*R* = 784 Ω), sweeping the voltage results in the diagram shown in Figure 3e. This figure represents a 2D map tracking the position of the dips for both rings as the voltage is increased. For a voltage of 6 V, the resonance matching condition for both rings is fulfilled. The Supporting Information includes a similar plot for a device connected in series, displaying similar behavior but requiring a higher operation voltage.

### Cladding Lift-off for Thermal Isolation

In standard methods, the cladding is deposited on the whole sample, making it challenging to place optical devices close to each other due to thermal crosstalk. Some approaches have shown that, to reduce the thermal crosstalk, the cladding between devices can be etched^61−64^ or predictive models can be developed to control their overall response.^65−67^ The possibility of defining the cladding using lift-off significantly reduces the design and fabrication complexity.

**Figure 4:**
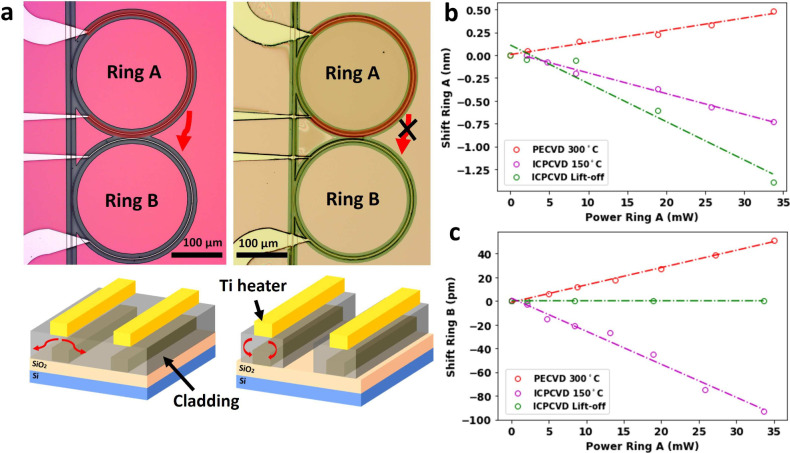
(a) Optical image (top view) of ring resonator devices fabricated by using continuous cladding (left) and cladding delimited with lift-off (right). Red arrows denote thermal crosstalk from the microheater of *R* = 1.9 kΩ. (b) Detuning as a function of power consumed in ring A fabricated using PECVD (red), ICPCVD (purple), and cladding lift-off (green). (c) Shift of the adjacent ring due to thermal crosstalk as a function of power consumed in the microheater for PECVD (red), ICPCVD (purple), and cladding lift-off (green).

Due to the low processing temperatures involved in ICPCVD we fabricated a cladding limited to a region of 4 μm around the waveguide and studied the thermal response. For reference, we also fabricated ring resonators with standard PECVD cladding. Both ring resonators have a 10 μm separation between adjacent waveguides and dimensions similar to those in previous sections. Figure 4a shows optical images of the devices together with a schematic side view of the cladding. To determine the thermal crosstalk between adjacent rings, we varied the power dissipated in the heater of ring A (*R* = 1.9 kΩ) and observed the thermal response of both rings A and B (Figure 4a). In the case of standard PECVD cladding, the thermal responses of rings A and B are 18.5 pm/mW (red line in Figure 4b) and 1.5 pm/mW (red line in Figure 4c), respectively. Similar to PECVD, for continuous ICPCVD cladding, the thermal responses of rings A and B are −22.7 pm/mW (purple line in Figure 4b) and −2.5 pm/mW (purple line in Figure 4c), respectively. By using an ICPCVD cladding with the lift-off method, we improve the performance of our device in two ways: we increase the thermal response in ring A (42 pm/mW, green line in Figure 4b) and decrease the thermal response in ring B (no thermal shift visible within a free spectral range, green line in Figure 4c). Therefore, we can thermally isolate two ring resonators placed 10 μm apart by depositing an ICPCVD cladding with the lift-off method, which is not feasible in standard PECVD claddings (see the Supporting Information for the measured data). Note that the heating efficiency of the on-chip microheaters falls outside the scope of this study, and the geometry of the heaters and thickness of the cladding can be modified to significantly raise the ratio between heat generation and power consumption and optimize the reconfiguration time.^68−72^

## Conclusions

We demonstrated, for the first time, the use of ICPCVD silicon dioxide claddings deposited at low temperatures to achieve positive, negative, and athermal thermo-optic devices on a single chip with large thermal tunability across several photonic platforms such as amorphous silicon carbide, silicon nitride, and silicon-on-insulator. Most importantly, we fabricated both passive and active components. Our approach opens up the possibility for the fabrication of low-power photonic configurations such as Mach–Zehnder interferometers, single-heater CROW optical devices, and highly sensitive temperature sensors that could be easily integrated with current electronic and photonic technologies. Additionally, we showed that the low-temperature fabrication scheme allows thermal isolation of the optical devices to compensate for the high thermal shifts and to increase the photonic integration densities. This study presents a phenomenological approach to tuning the thermal properties of optical devices. While preliminary research into the root cause of the effect is presented in the Supporting Information, more comprehensive studies are needed to fully unravel the mechanism behind the exciting findings that are reported here. Measurements of film stress, surface morphology, and anisotropy ratio of the cladding indicate that these properties could be the main mechanism behind this effect. Nevertheless, to model these devices, it is important to understand other contributions such as the composition and density of the films, hydrogen, nitrogen, or dopant incorporation, specific mechanical constants of the deposited films (Young modulus and Poisson constant), the strain between the layers, and how the device geometry can affect the thermal tunability. With further improvements, we foresee the use of these configurations for novel photonic architectures and widely tunable photonic circuits.

## Methods

### Deposition Recipes for Silicon Dioxide

Using PECVD we deposited SiO_2_ in a mixture of 8.5 sccm SiH_4_, 710 sccm N_2_O, and 165 sccm N_2_ with a plasma power of 20 W and chamber pressure of 1000 mTorr. The average deposition rates for all recipes were around 70 nm/min. The deposition of SiO_2_ claddings using ICPCVD was done using a mixture of 16 sccm of SiH_4_ and 60 sccm of N_2_O with a plasma power of 1300 W and a chamber pressure of 8 mTorr. The average deposition rates for all recipes were around 60 nm/min.

### Device Fabrication

The fabrication of a-SiC optical devices was performed following our previous work.^27^ The development of the electron beam resist was performed in pentyl-acetate, MBIK:IPA (1:1), and IPA. For the etching of a-SiC and silicon-on-insulator, the plasma power was set to 20 W with 13.5 sccm of SF_6_ and 3.5 sccm of O_2_ at a chamber pressure of 8 μbar. LPCVD silicon nitride devices were fabricated on 380 nm thick films. Etching of these devices was performed at 20 °C in a mixture of CHF_3_ and O_2_. The excess e-beam resist was removed by oxygen plasma cleaning (200 sccm O_2_ at 50 W) for 5 min. The lift-off of silicon dioxide was performed by using PMMA 950K A11 with a thickness of 2.2 μm. The resist was spin-coated at a rate of 2500 rpm/min and baked for 10 min at a temperature of 175 °C. To remove resist residues after development, we performed an oxygen plasma cleaning for 60 s (200 sccm O_2_ at 50W). The lift-off was done by immersing the samples in acetone and heating the solution to 53 °C. Silicon dioxide claddings were deposited via ICPCVD or PECVD with a thickness of 2 μm. The contact pads were made of 80 nm titanium and 10 nm gold and patterned using lift-off with a PMMA A6-950 K resist with a thickness of 1 μm.

### Device Characterization

For device characterization, we used a grating coupler configuration. The light from the laser source was coupled to a polarization-maintaining fiber by using a U-Bench. The polarization going to the device was controlled with a free-space polarizer. Paddle polarizers (FPC560) were used to align the polarization of the laser source with the free-space polarizer (FBR-LPNIR). The thermal response of the devices was recorded using an optical spectrum analyzer (OSA Yokowaga AQ6374) and a tunable laser (Photonetics TUNICS-PRI 3642 HE 15) with a powermeter (818-IR Newport, linearity 0.5%). The sample was placed in a PCB using thermally conductive silver paste and heated with a thermal element in the sample stage with a temperature PIC controller (see the Supporting Information). Several graphs for the different platforms show the shift in resonance wavelength as a function of the stage temperature and are represented in the Supporting Information. Electrical tuning of the devices was done with microheaters of 80 nm thick titanium (Ti) on top of the 2 μm SiO_2_ cladding. The voltage was swept using a programmable voltage supply (RIGOL model DP832A) and a customized MATLAB script. The propagation losses were calculated using the intrinsic quality factor and group index of the devices following previous studies.^27,73^

## Data Availability

The data and fabricated samples supporting this study are available from the corresponding authors for further analysis upon reasonable request. Additional content is available at https://arxiv.org/abs/2407.08480.

## Supporting Information Available

Detailed information about film characterization, optical setup, summarized data for each device, passive device configurations, strain release experiments, representative spectra for the different platforms, additional measurements for CROW devices, thermal crosstalk spectra, and electron microscope image of the lifted-off cladding (PDF)
