# Supplementary material for: Magic Silicon Dioxide for Widely Tunable Photonic Integrated Circuits
Source: ACS Photonics. 2025 Feb 5;12(3):1321–8. doi: 10.1021/acsphotonics.4c01373 (PMC11926958; doi:10.1021/acsphotonics.4c01373)
Supplement: Supplementary file 1 — ph4c01373_si_001.pdf [file ph4c01373_si_001.pdf]

Supplementary information: Magic silicon dioxide for widely tunable photonic integrated circuits

Bruno Lopez-Rodriguez[1], Naresh Sharma[1], Zizheng Li[1], Roald van der Kolk[1],
Jasper Van Der Boom[1], Thomas Scholte[1], Jin Chang[2], Simon Gröblacher[2] and Iman Esmaeil Zadeh[1]

[1]Department of Imaging Physics (ImPhys), Faculty of Applied Sciences, Delft University of Technology, Delft 2628 CJ, The Netherlands
[2]Department of Quantum Nanoscience, Faculty of Applied Sciences, Delft University of Technology, Delft 2628 CJ, The Netherlands

E-mail: b.lopezrodriguez@tudelft.nl

Table of Contents

1. Film Characterization……2

1.1. Film stress……2

1.2. Atomic Force Microscopy……2

1.3. Ellipsometry……3

2. Optical setup and summarized data……3

2.1. Schematic of measurement setup……3

2.2. Optical properties of fabricated devices……4

3. Passive devices……7

4. Strain release……7

4.1. Deposition of PECVD films……7

4.2. Low temperature cladding……8

4.3. Temperature stability……8

5. Representative spectra for the different platforms……9

5.1. Amorphous silicon carbide……9

5.2. Silicon nitride……11

5.3. Silicon-on-insulator……12

6. Coupled Ring Optical waveguide (CROW) devices……14

7. Thermal crosstalk……15

7.1. PECVD Continuous film……15

7.2. ICPCVD Continuous film……16

7.3. ICPCVD lift-off cladding……16

8. Ramp-up and ramp-down measurements……17

9. Stability measurements of the optical setup……18

1. Film characterization

We performed stress measurements, atomic force microscopy and ellipsometry of the deposited silicon dioxide films and the results are found in this section. Overall, these measurements are an indication of the different properties that could be affecting the thermal expansion and conduction of the films. Stress measurements indicate overall stress in the film and not local stress on specific devices, which might be higher around the waveguide region due to anisotropy differences. AFM measurements reveal the grain sizes, surface roughness and deposition topography (skew) of the silicon dioxide claddings. As discussed in the main manuscript and supported by literature studies, these parameters affect the thermal expansion and conduction properties of a material. Ellipsometry data provides refractive index and an estimation of the density of the films, showing similar optical properties among all deposited films independent of deposition temperature or chamber pressure.

1.1. Film stress

We measured the stress of amorphous silicon carbide and silicon dioxide films using FLX-2320-S Thin Film Stress Measurement system from Toho Technology. Silicon carbide films were deposited on top of thermally oxidized silicon wafers (525 µm) with oxide thickness of 8 µm. To characterize the silicon dioxide films they were deposited on bare silicon wafers. The data is summarized in **table S1**.

Technique and condition	Stress (MPa)
a-SiC PECVD 300°C	-50
a-SiC ICP 150°C	-300 to -500
SiO_2 ICP 30°C	10
SiO_2 ICP 150°C	10.4
SiO_2 ICP 300°C	-22.9
SiO_2 PECVD 300°C	-20

Table S1. *Stress data for a-SiC and SiO_2 deposited using PECVD and ICPCVD techniques.*

1.2. Atomic Force Microscopy

We performed AFM scans of the deposited silicon dioxide films via PECVD and ICPCVD at different temperatures and retrieved surface morphology data, mainly surface roughness and skewness. The latter measures whether the surface has more deep valleys (negative skew) or protruding narrow peaks (positive). Three examples of AFM scans taken at different temperatures and techniques can be seen in **fig.S1** and results can be found in **table S2** with varying temperatures.

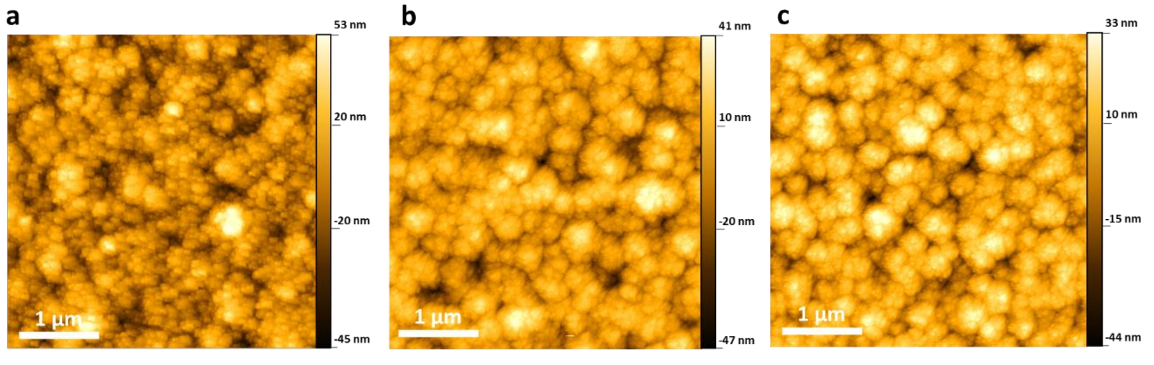

Figure S1. *Atomic Force Microscope scans of silicon oxide deposited **a)** with PECVD at 300°C, **b)** with ICPCVD at 30°C and **c)** with ICPCVD at 150°C.*

1.3. Ellipsometry

To characterize the refractive index of the silicon dioxide films deposited via ICPCVD and PECVD, we used Woollam M-2000 spectroscopic ellipsometer and fitted the corresponding data with a Cauchy model for both PECVD and ICPCVD at different deposition temperatures (**Fig.S2a**). For ICPCVD, we also performed ellipsometry for different chamber pressures (**Fig.S2b**). **Table S2** summarizes the thickness, refractive index, surface roughness and skew obtained for the different films.

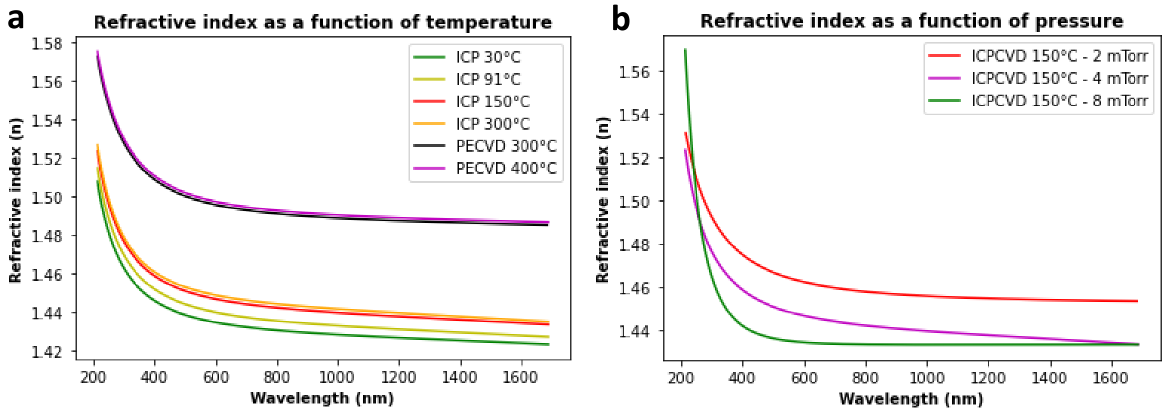

Figure S2. *Ellipsometry data for the refractive index as a function of wavelength for PECVD and ICPCVD Silicon Dioxide films deposited at different **a)** temperatures and **b)** chamber pressure.*

Parameter / Technique	Thickness (nm)	Refractive index	Surface roughness (RMS nm)	Skew
**ICP 30°C**	2786.58	1.433	10.19 ± 1.34	-0.99
**ICP 91°C**	2746.93	1.443	---	---
**ICP 150°C**	2641.77	1.445	9.55 ± 1.28	-0.09
**ICP 300°C**	2607.79	1.447	11.49 ± 1.66	-0.12
**PECVD 300°C**	3101.33	1.494	12.11 ± 1.91	0.19
**PECVD 400°C**	3284.44	1.496	10.75 ± 1.49	0.01

Table S2. *Data for silicon dioxides deposited with ICPCVD and PECVD at different temperatures representing film thickness, refractive index, surface roughness and AFM skew. (---) refers to not measured.*

2. Characterization setup and summarized data

2.1. Characterization setup

An schematic of the characterization setup described in the main manuscript is shown in **fig. S3a** with a picture of the optical setup in the lab shown in **fig.S3b**.

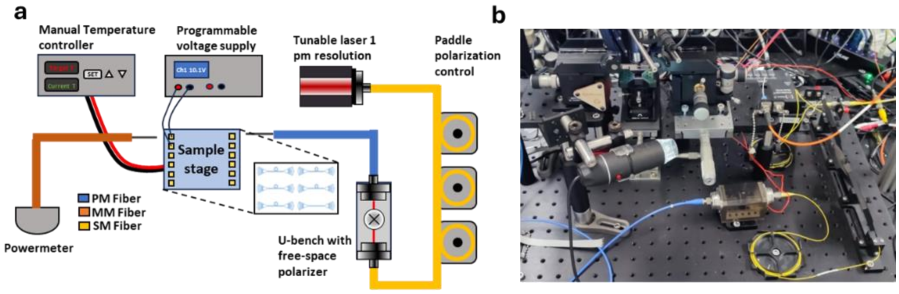

Figure S3. a) *Schematic of the optical setup used for the measurements and **b)** full optical setup in the lab.*

2.2. Optical properties of fabricated devices

For the silicon dioxide claddings deposited at different temperatures via ICPCVD and PECVD on a-SiC, SiN and SOI, we summarize in **table S3** the refractive index, free spectral range and corresponding group index of optical devices with dimensions stated in **table S5**.

	SiO_2 T (°C)	n oxide	a-SiC		SiN		SOI	
			FSR (nm)	n_g	FSR (nm)	n_g	FSR (nm)	n_g
**PECVD**	200	---	1.32	2.41	---	---	0.96	3.32
	300	1.49440	1.30	2.45	1.49-1.55	2.14*	0.96	3.32
	400	1.49605	1.22	2.61	---	---	---	
**ICP**	30	1.43336	1.34	2.38	1.83	1.74	0.87	3.66
	75	---	---	---	---	---	0.90	3.54
	150	1.44543	1.32	2.41	1.69-1.59	1.89*	0.86	3.71
	300	1.49605	1.30	2.45	1.80	1.68	0.86	3.71
	400	---	1.31	2.43	1.71-1.31	1.86*	0.87	3.66

Table S3. *Optical properties of the devices fabricated using different oxides with PECVD and ICPCVD at different temperatures. All the free spectral ranges are taken from the spectral measurement done at room temperature. *The corresponding group index is calculated assuming the FSR of the first mode. (---) refers to samples not fabricated.*

For silicon dioxide claddings deposited via ICPCVD on a-SiC platform at a fixed deposition temperature of 150°C, we summarize in **table S4** the free spectral range and group index of the fabricated devices.

**Oxide pressure (mTorr)**	1.5	2	2.5	4	6	8	10	12	16
**FSR (nm)**	1.33	1.32	1.34	1.35	1.36	1.32	1.43	1.34	1.40
**n_g**	2.40	2.41	2.38	2.36	2.34	2.41	2.23	2.38	2.28

Table S4. *Optical properties of the devices made with Silicon Dioxide deposited via ICPCVD at 150°C varying the chamber pressure.*

To determine the effective thermo-optic coefficient, we simulated the mode profile with the selected dimensions using Ansys Lumerical MODE solutions for the different platforms. The table below summarizes information about width, thickness, ring radius, refractive index and obtained effective index. In **table S6** we summarize the mode overlap factor for the different platforms using the simulation results in **table S5**.

Material	Width / Thickness (nm)	Ring radius (µm)	n	n_{eff}
ICPCVD a-SiC 150°C	800 / 271	120	2.67	1.937
SOI	700 / 220	120	3.44	2.565
Silicon Nitride	1000 / 368	120	2	1.633

Table S5. *For the studied ring resonators summary of material platform, waveguide dimensions, ring radius and effective index calculated using FDTD (Ansys Lumerical MODE solutions).*

Material	Overlap waveguide (%)	Overlap cladding (%)	Overlap substrate (%)
ICPCVD a-SiC 150°C	71.4856	13.7907	14.7237
SOI	79.4482	9.644	10.9078
Silicon Nitride	63.4867	20.6400	15.8733

Table S6. *For the studied ring resonators mode fill factors calculated using FDTD (Ansys Lumerical MODE solutions).*

Table S7 summarizes the wavelength shifts in pm/°C and the corresponding effective thermos-optic coefficient for the different claddings deposited on a-SiC, SiN and SOI.

SiO_2 T (°C)		a-SiC		SiN		SOI	
Technique		Shift (pm/°C)	TOC_{eff} (10^{-5})	Shift (pm/°C)	TOC_{eff}(10^{-5})	Shift (pm/°C)	TOC_{eff}(10^{-5})
PECVD	200	16.9 ± 0.4	2.12	---	---	17.10 ± 0.7	3.00
	300	32.4 ± 1.0	4.63	14.29 ± 0.4	1.41	38.0 ± 0.4	7.47
	400	31.4 ± 0.3	4.78	---	---	---	---
ICPCVD	30	---	---	-157.3 ± 22.1	-18.16	13.9 ± 2.2	2.62
	75	-165.9 ± 9.2	-25.96	---	---	-94.6 ± 9.6	-22.28
	150	-90.0 ± 8.1	-14.52	-86.4 ± 24.1	-11.03	---	---
	300	-96.7 ± 7.9	-15.80	-106.0 ± 7.9	-11.99	4.8 ± 0.7	0.48
	400	-68.1 ± 23.5	-11.19	-36.94 ± 5.8	-4.97	17.4 ± 0.8	3.45

Table S7. *For a-SiC, SiN and SOI optical ring resonators, thermal shift in pm/°C and effective thermo-optic coefficient for different oxide temperatures and techniques. (---) refers to samples not fabricated/non-functional devices. As a reference, PMMA on SiN has thermal tunability of -51 pm/°C.*

For the ICPCVD silicon dioxide claddings deposited at a temperature of 150°C and varying the chamber pressure, we summarize in **table S8** the wavelength shift in pm/°C and the corresponding effective thermos-optic coefficient.

Oxide pressure (mTorr)	1.5	2	2.5	4	6	8	10	12	16
Shift (pm/°C)	27.5	10.2	21.6	-52.1	-46.5	-62	-78.5	-84.5	-117.7
STD ± Δσ (pm/°C)	1.6	1.1	1.5	2.6	5.5	8	7	0.4	7.1
TOC_{eff} (10^{-5})	3.75	1.09	2.81	-8.44	-7.53	-10.02	-11.18	-13.47	-17.85

Table S8. *For a-SiC optical ring resonators, thermal shift in pm/°C and effective thermo-optic coefficient with an ICPCVD oxide cladding deposited at 150°C at different chamber pressures.*

To determine the losses introduced by the different claddings deposited at different deposition temperatures and pressures on a-SiC optical devices, we summarized the main parameters of the analysed resonance dip together with the optical losses in dB/cm in **table S9**.

Temperature (°C)	30	150	300
Full-Width at Half-Maximum (FWHM - pm)	14.50	11.88	13.77
Wavelength (nm)	1549.73	1548.50	1549.87
Transmission (au)	0.367	0.412	0.460
Loaded quality factor	107,000	130,000	113,000
Intrinsic quality factor	133,000	158,000	134,000
Group index	2.378	2.41	2.45
Loss (dB/cm)	3.15	2.68	3.22

Table S9. *Optical data for different ICPCVD Silicon Dioxide at a chamber pressure of 8 mTorr and different deposition temperatures of specific transmission dips.*

We also deposited one of the ICPCVD claddings at a temperature of 300°C with a chamber pressure of 12 mTorr on a-SiC, corresponding to the wavelength spectra reported in the main manuscript (**fig.2c**). The data to determine the losses can be found in **table S10** has been taken from the spectra at room temperature (27°C).

T (°C)	P (mTorr)	FWHM (pm)	Wavelength (nm)	T (au)	Q_{load}	Q_{int}	n_g	Loss (dB/cm)
300	12	22.45	1550.22	0.265	69,000	91,000	2.41	4.74

Table S10. *Optical data for ICPCVD Silicon Dioxide deposited at a temperature of 300°C and chamber pressure of 12 mTorr of a specific transmission dip.*

Table S11 summarizes main parameters of the measured resonance for a-SiC devices depositing silicon dioxide claddings via ICPCVD at a deposition temperature of 150°C and varying chamber pressure from 2.5 mTorr to 16 mTorr.

Pressure (mTorr)	2.5	8	10	16
Full-Width at Half-Maximum (FWHM)	13.07	11.88	18.80	30.27
Wavelength (nm)	1548.32	1548.50	1548.38	1550.4
Transmission (au)	0.329	0.412	0.363	0.250
Loaded quality factor	118,000	130,000	82,360	51,220
Intrinsic quality factor	150,000	158,000	103,000	68,000
Group index	2.38	2.41	2.23	2.28
Loss (dB/cm)	2.79	2.69	3.81	5.90

Table S11. *Optical data for ICPCVD Silicon Dioxide deposited at a temperature of 150°C and different chamber pressures of a specific transmission dip.*

Fig. S4 shows a graphical representation of the change of free-spectral range, quality factor and refractive index for ICPCVD films deposited at different temperatures and pressures. **Fig.S4c** also shows the refractive index of silicon dioxide claddings deposited via PECVD at 300°C and 400°C.

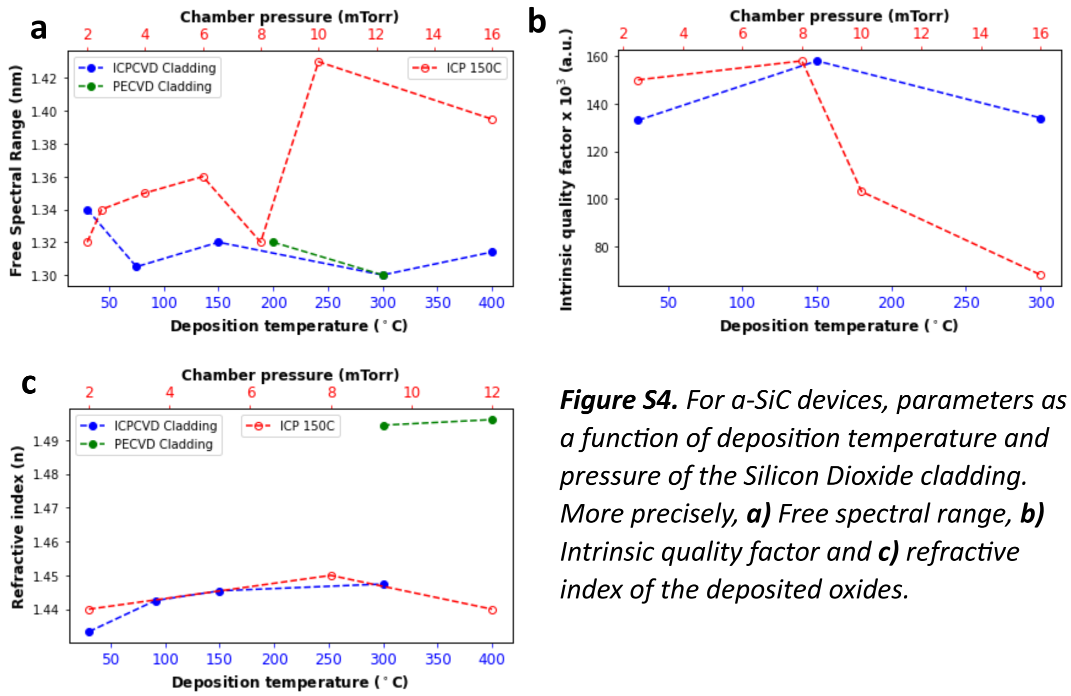

Figure S4. *For a-SiC devices, parameters as a function of deposition temperature and pressure of the Silicon Dioxide cladding. More precisely,* ***a)*** *Free spectral range,* ***b)*** *Intrinsic quality factor and* ***c)*** *refractive index of the deposited oxides.*

3. Passive devices

We demonstrate two different passive configurations of optical devices. **Fig.S5a** shows the resulting spectra two ring resonators connected in series with positive and negative claddings as the temperature of the sample stage is raise from 20°C to 35°C in steps of 2°C. **Fig.S5b** shows an optical microscope image of a Mach-Zehnder interferometer where one of the arms is covered with a cladding deposited via ICPCVD at a temperature of 150°C and chamber pressure of 8 mTorr. Raising the stage temperature vary the relative phase between each arm and the intensities at different outputs.

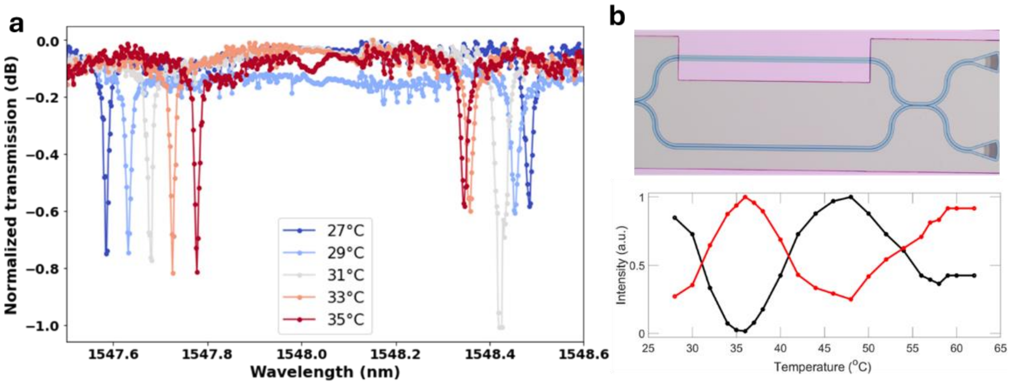

***Figure S5. a)** Spectra of ring resonators at different temperatures fabricated on the same chip with shared bus waveguide and claddings presenting bidirectional thermal response and **b)** Optical microscope image of a Mach-Zehnder interferometer covered with ICPCVD SiO_2 cladding in one of the arms and intensity as a function of temperature for the two output ports.*

4. Strain release

4.1. Deposition of PECVD films

As an experiment to determine the effect in the thermal tunability when depositing other films with opposite thermal expansion, we deposited PECVD claddings on top of the ICPCVD cladded devices deposited at 150°C. The resonance wavelength position as a function of the stage temperature for the different configurations is depicted in **fig.S6**. It is observed that the dominant shift is similar to the one introduced by only using PECVD cladding and it cannot be attributed to annealing effects in the films as shown in section 4.3.

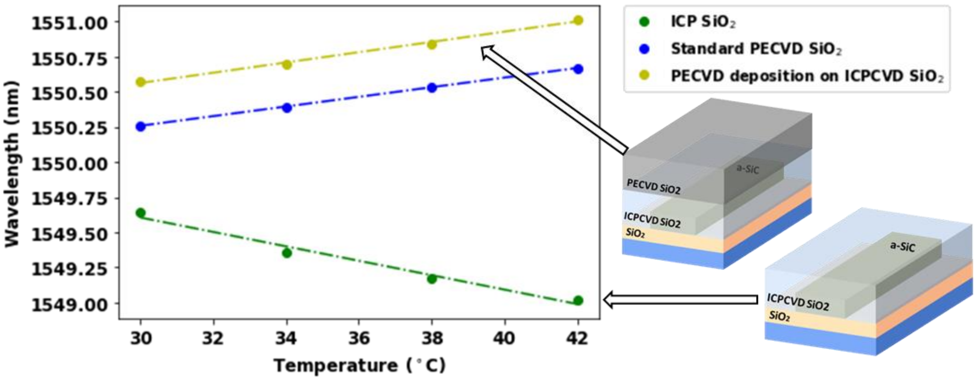

***Figure S6.** Wavelength shift as a function of temperature for optical ring resonators covered with ICPCVD SiO_2, Standard PECVD SiO_2 and PECVD on top of ICPCVD oxide.*

4.2. Low temperature cladding

We deposited a silicon dioxide cladding via ICPCVD at 30°C on silicon nitride devices and the wavelength spectra as a function of the stage temperature between 27°C and 35°C is shown in **fig.S7**. When measuring the thermal response of the resonance, we observed that for stage temperatures higher than 33°C, there is a non-linear jump and the thermal tunability becomes lower. We attribute this effect to strain release between the core and the cladding that causes a decrease in the thermal tunability of the optical devices.

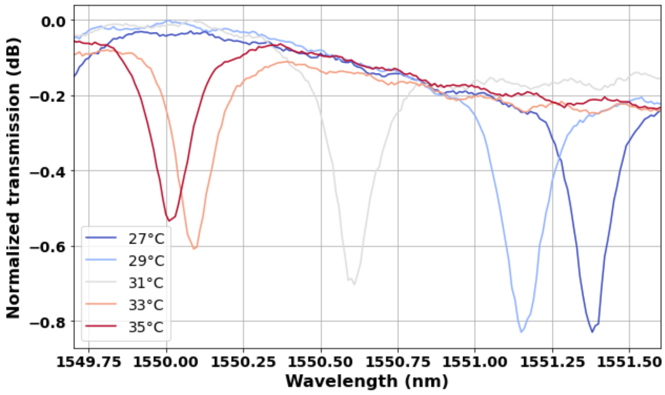

Figure S7. *Wavelength spectra around 1550 nm for ICPCVD 30°C Silicon Dioxide cladding on a SiN device as a function of temperature between 27°C and 35°C in steps of 2°C.*

4.3. Temperature stability

On films deposited via ICPCVD at a temperature of 150°C and chamber pressure of 8 mTorr, we also performed high temperature processing of the devices to investigate possible changes in the thermo-optic shift. The temperature range was done in incremental steps from 200°C to 400°C during 1h and the resulting position of the resonance dip as a function of stage temperature for the different processing temperatures is shown in **fig.S8**. We observed no difference in the thermal tunability.

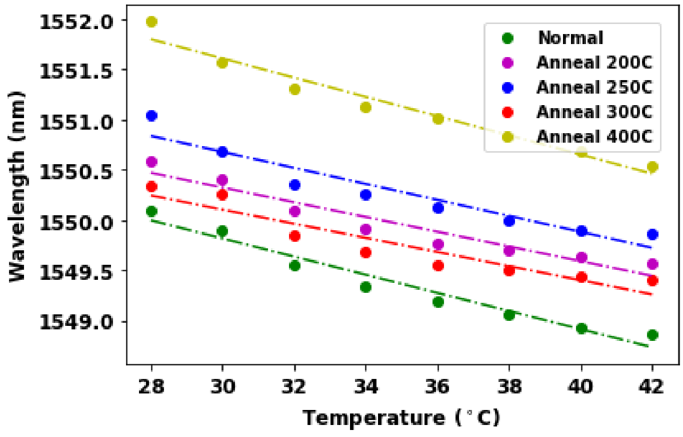

Figure S8. *Wavelength shift as a function of the stage temperature of the selected resonance dip for different temperatures, from the standard devices (green) to annealing at 400°C (yellow).*

5. Representative spectra for the different platforms

Below we include representative data and spectra for the wavelength shift of devices on amorphous silicon carbide, silicon nitride and silicon-on-insulator using different deposition conditions, mainly chamber pressure and deposition temperature.

5.1. Amorphous Silicon Carbide

In **fig.S9** we represent the wavelength shift as a function of stage temperature for ICPCVD silicon dioxide claddings deposited at 150°C and chamber pressures of 1.5 mTorr and 12 mTorr corresponding to negative and positive thermal tunability. **Fig.S10** shows the resulting wavelength spectra taken at different stage temperatures and linear fitting to obtain the wavelength tunability for a-SiC devices with silicon dioxide ICPCVD claddings deposited at 30°C and chamber pressure of 8 mTorr. This device presents the largest shift in wavelength in a stage temperature range between 27°C and 30°C.

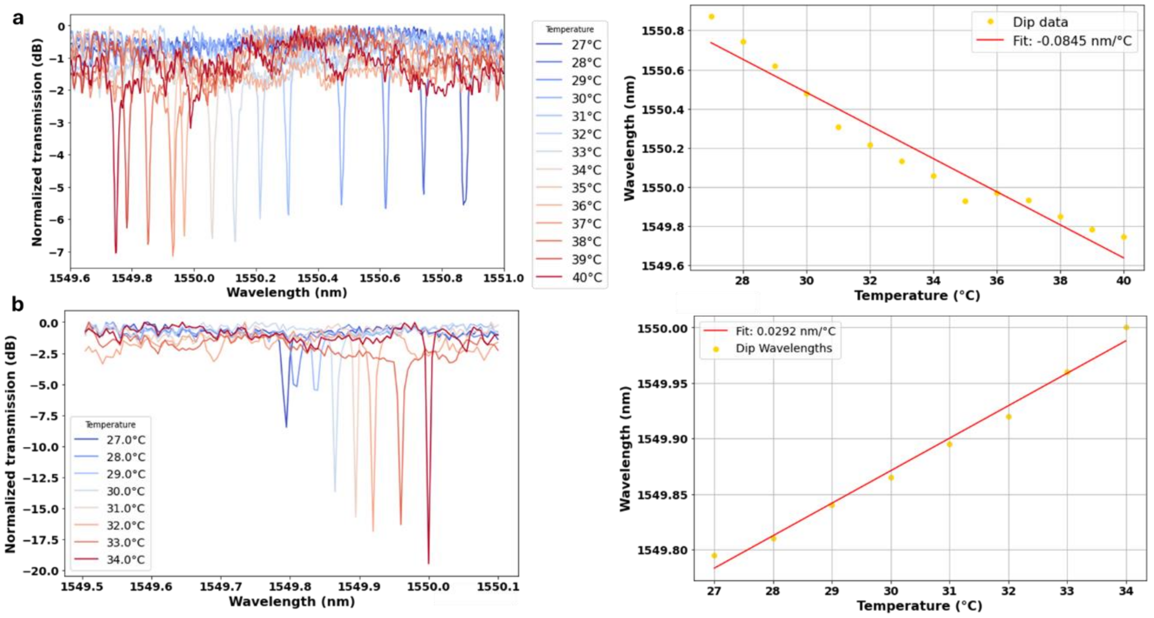

Figure S9. *Spectra taken at different temperatures for a-SiC ring resonators with SiO_2 cladding deposited via ICPCVD at 150°C and corresponding fitting for chamber pressure of* ***a)*** *12 mTorr and* ***b)*** *1.5 mTorr.*

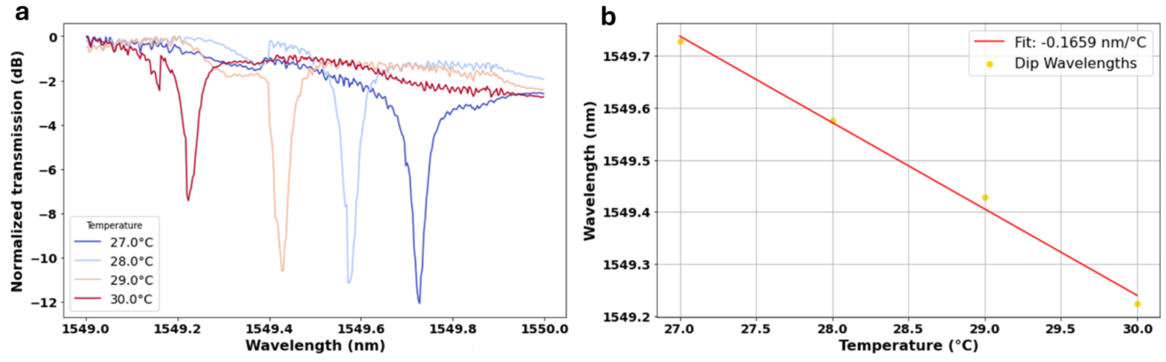

Figure S10. a) *Normalized spectra at different stage temperatures for an a-SiC optical device with ICPCVD SiO_2 cladding deposited via ICPCVD at 75°C (ramp up to 91°C) and chamber pressure of 8 mTorr and* ***b)*** *resonance dip position with linear fitting.*

We also include in **fig.S17** the wavelength spectra at different stage temperatures and corresponding resonance wavelength fitting of ICPCVD claddings deposited via ICPCVD at 150°C and varying the chamber pressure. The wavelength accuracy is limited by the step size of the wavelength sweeps (0.005 nm) and the accuracy of the laser (absolute wavelength accuracy of ±0.2 nm, tuning accuracy of ±0.02 nm and tuning repeatability of ±0.005 nm). Every time a scan is finished, the laser is set back to the initial wavelength and the limiting factor is the laser positioning accuracy. The measurements done with the optical spectrum analyser (for all the devices with SiN and SOI and a-SiC devices using PECVD cladding) show minimal standard deviation in the resonant dip position.

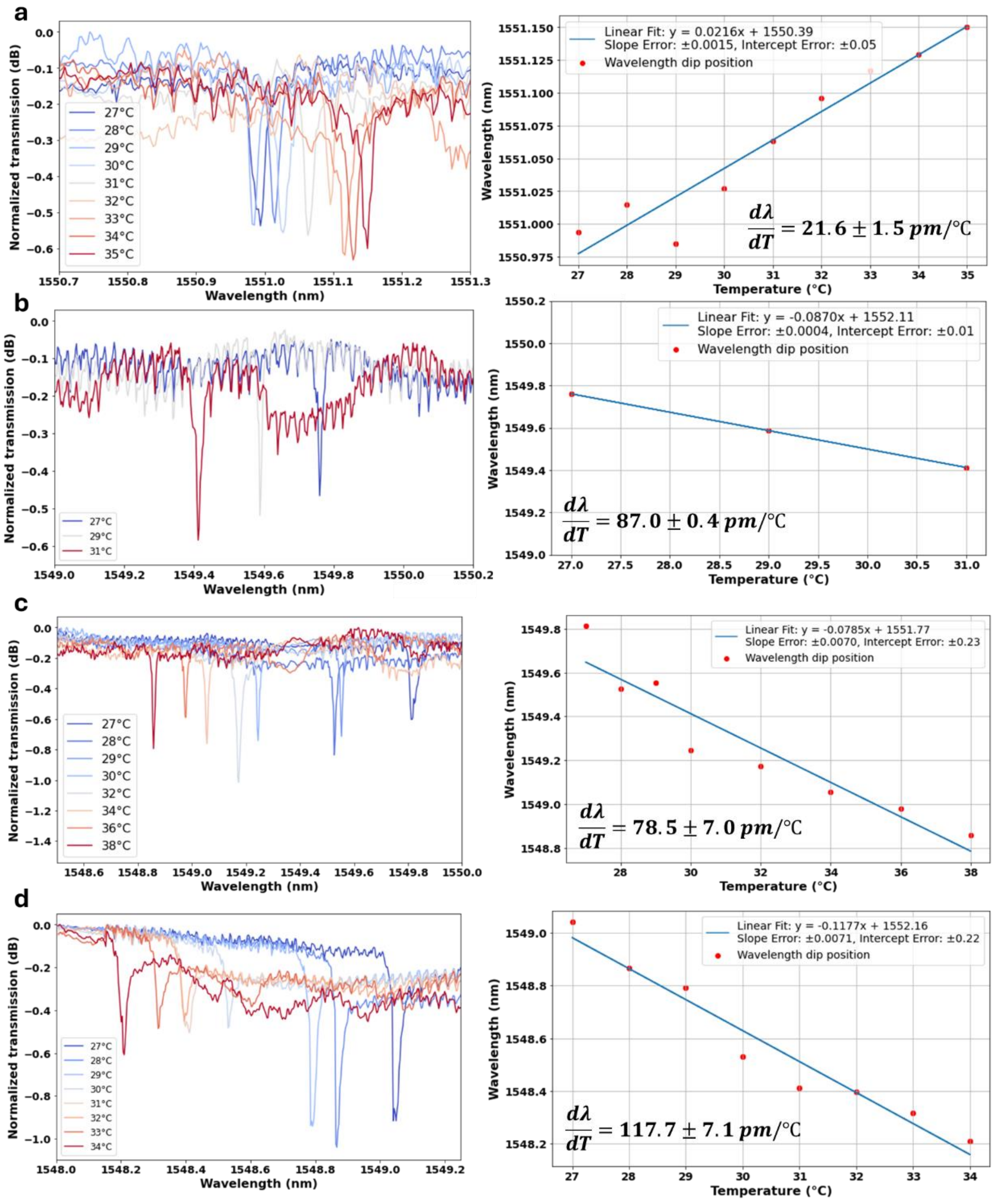

Figure S11. *Spectra taken at different temperatures for a-SiC ring resonators with* SiO_2 *cladding deposited via ICPCVD at 150°C and corresponding fitting for chamber pressure of* ***a)*** *2.5 mTorr,* ***b)*** *8 mTorr,* ***c)*** *10 mTorr and* ***d)*** *16 mTorr.*

The device that presented the highest negative shift for the a-SiC platform was fabricated using ICPCVD SiO_2 deposited at a temperature of 300°C and chamber pressure of 12 mTorr. The fitting of the resonance dip as a function of temperature is represented in **fig.S12** and corresponds to the data shown in **fig.2b** in the main manuscript.

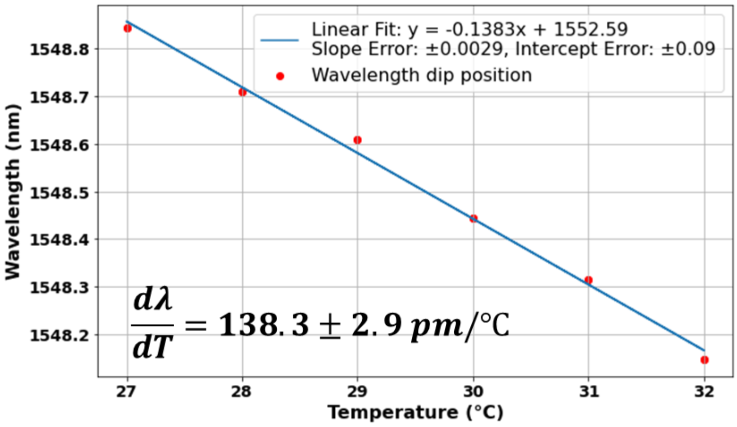

Figure S12. *For silicon dioxide deposited using ICPCVD at a temperature of 300°C and chamber pressure of 12 mTorr, wavelength position of the selected resonance dip position as a function of temperature together with the linear fitting.*

Attending to the systematic measurements done for ICPCVD claddings deposited at a temperature of 150°C and varying the chamber pressure on a-SiC optical devices, the athermal condition can be achieved for a chamber pressure of 3 mTorr. We deposited a silicon dioxide cladding on a-SiC with these conditions and measured the ring resonator in a temperature range between 27°C and 41°C in steps of 1°C. The resulting spectra is shown in **fig.13a** with the corresponding wavelength position as a function of the stage temperature in **fig.13b**. The thermal tunability between 27°C and 35°C obtained from fitting the data is 1.1 pm/°C . In the main article, the same data is represented in steps of 2°C resulting in a thermal tunability of 1.5 pm/°C.

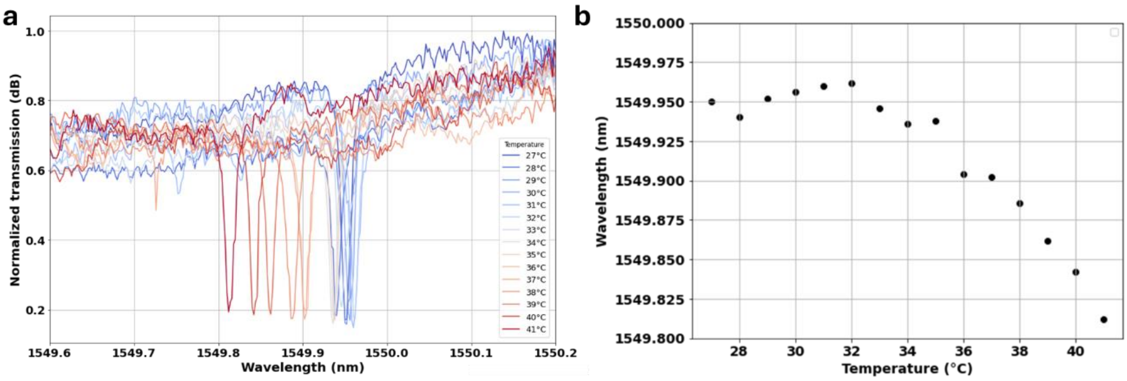

Figure S13. *For an a-SiC ring resonator with ICPCVD SiO_2 cladding deposited at 150°C and chamber pressure of 3 mTorr shown in figure 2 of the manuscript* ***a)*** *Normalized spectra taken at different temperatures with steps of 1°C and* ***b)*** *dip position as a function of temperature.*

5.2. Silicon Nitride devices

We deposited silicon dioxide claddings on silicon nitride devices via ICPCVD and PECVD at different temperatures and the resulting wavelength spectra as a function of stage temperature is found in **fig.S14.** As a reference, we also included the effect of a common electron beam resist PMMA on the thermal shift, known to give negative thermo-optic tunability.

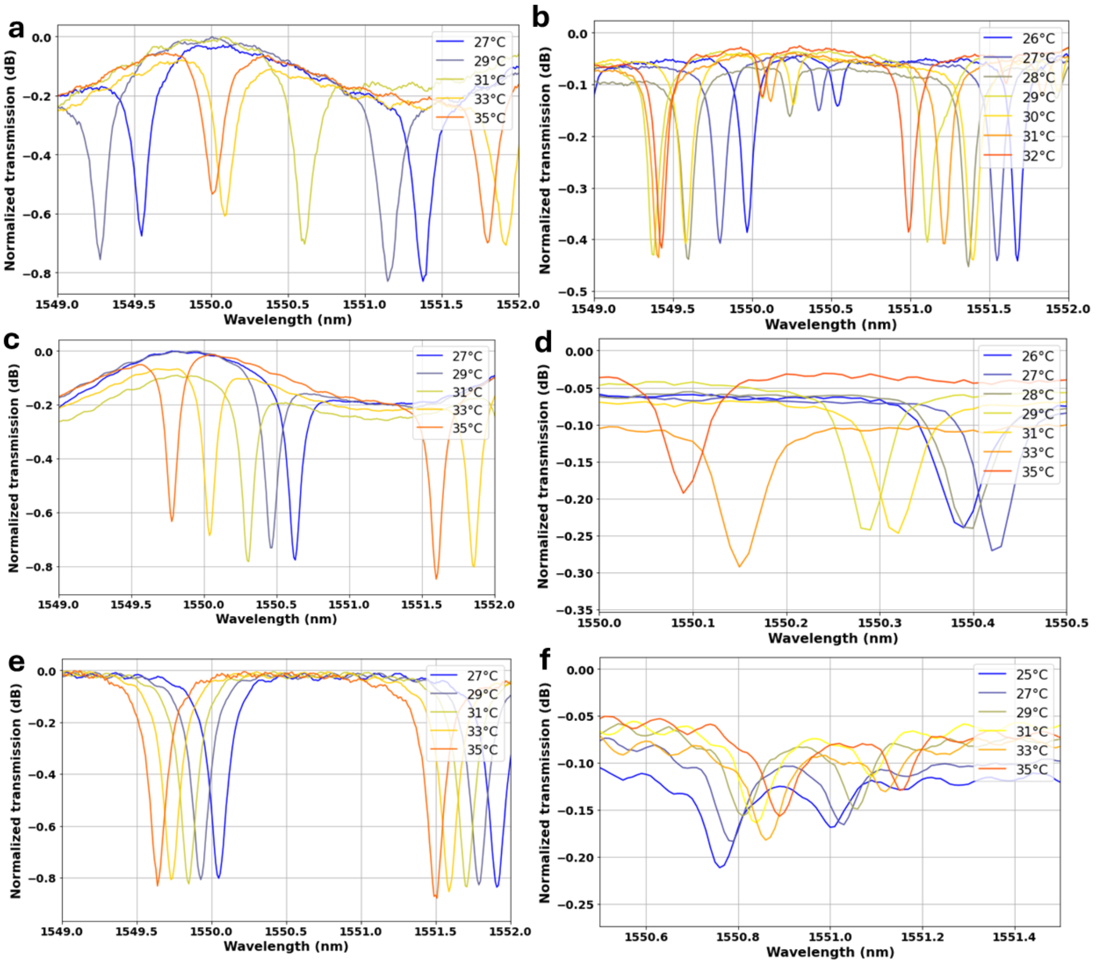

Figure S14. *Wavelength shift as a function of temperature for SiN device using ICPCVD SiO_2 deposited at* ***a)*** *30°C,* ***b)*** *150°C,* ***c)*** *300°C,* ***d)*** *400°C,* ***e)*** *PMMA (-51 pm/°C) and* ***f)*** *PECVD SiO_2 deposited at 300°C.*

5.3. Silicon-On-Insulator devices

We deposited SOI optical devices using PECVD at temperatures of 200°C and 300°C and ICPCVD at a temperature of 75°C (**fig.S15**) and the wavelength spectra as a function of temperature is shown in **fig.S15**. We also deposited silicon dioxide via ICPCVD at 300°C and chamber pressure of 8 mTorr resulting in a thermal tunability of 5.5 pm/°C. The wavelength spectra as a function of the temperature and the corresponding fitting for the two resonances separated one free spectral range is shown in **fig.S16**.

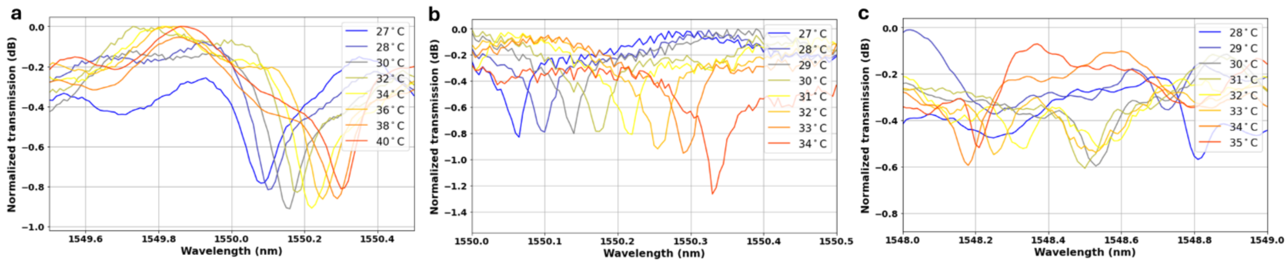

Figure S15. *Wavelength shift as a function of temperature for an SOI device with SiO_2 claddings deposited using* ***a)*** *PECVD at 200°C,* ***b)*** *PECVD at 300°C and* ***c)*** *ICPCVD at 75°C.*

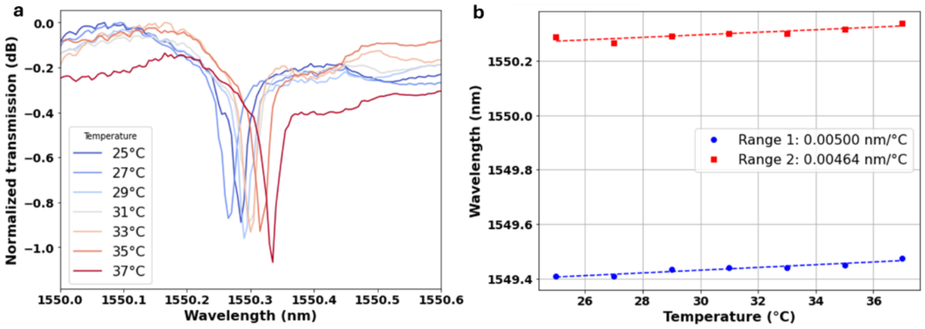

Figure S16. a) *Transmission spectra for a Silicon-On-Insulator device with SiO_2 cladding deposited via ICPCVD at 300°C and chamber pressure of 8 mTorr and* ***b)*** *corresponding fitting of the two dips separated by a free spectral range.*

6. Coupled-Resonator Optical Waveguide (CROW) devices

We fabricated one sample with two CROW devices using positive and negative claddings and metal micro-heaters. We measured this devices by sweeping the voltage and recorded the spectra.

6.1. CROW device 1

In the same configuration as the device shown in the main manuscript, we measured another device connected in parallel as depicted in **fig.S17a** in a voltage range of 0V to 12V in steps of 0.5V. **Fig.S17b** shows that the resonance condition can be achieved at a voltage of 9.5V. **Fig.S17c** shows wavelength spectra taken at different voltages of 0V, 7.5V and 9.5V (resonance condition).

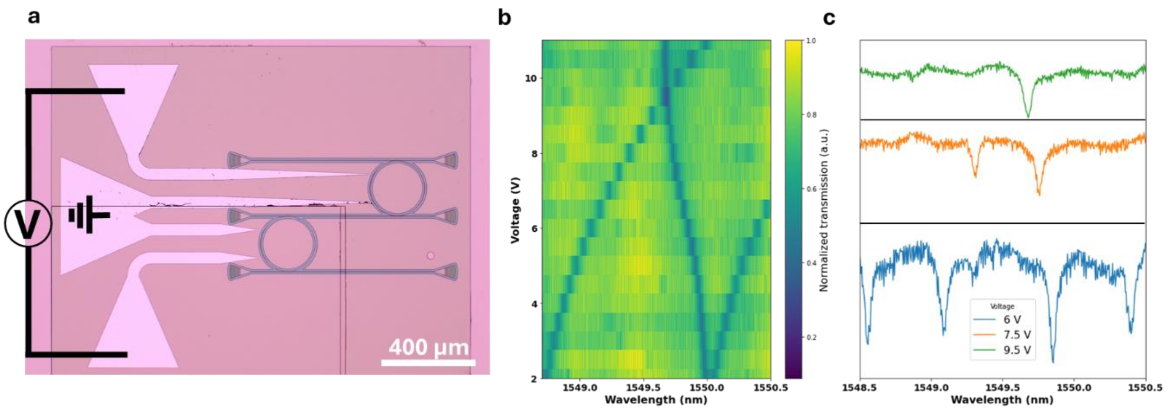

Figure S17. a) *Optical microscope image of device 1 with two ring resonators connected with a middle waveguide to track the resonance dips in parallel connection configuration.* ***b)*** *Wavelength intensity spectra as a function of voltage applied and* ***c)*** *Wavelength spectra for 6V (blue), 7.5V (orange) and 9.5V (green). The spectra are separate between each other.*

6.2. CROW device 2

For the same device as the one shown in the manuscript (**fig.3b-c**) and in the same configuration (heaters connected in parallel), we did a coarse scan of the voltage in steps of 1 V from 0 to 13 V. A 2D mapping of the transmitted intensity as a function of the wavelength is shown in **fig.S18** together with the specific spectra taken at different voltages of 0V, 3V and 6V.

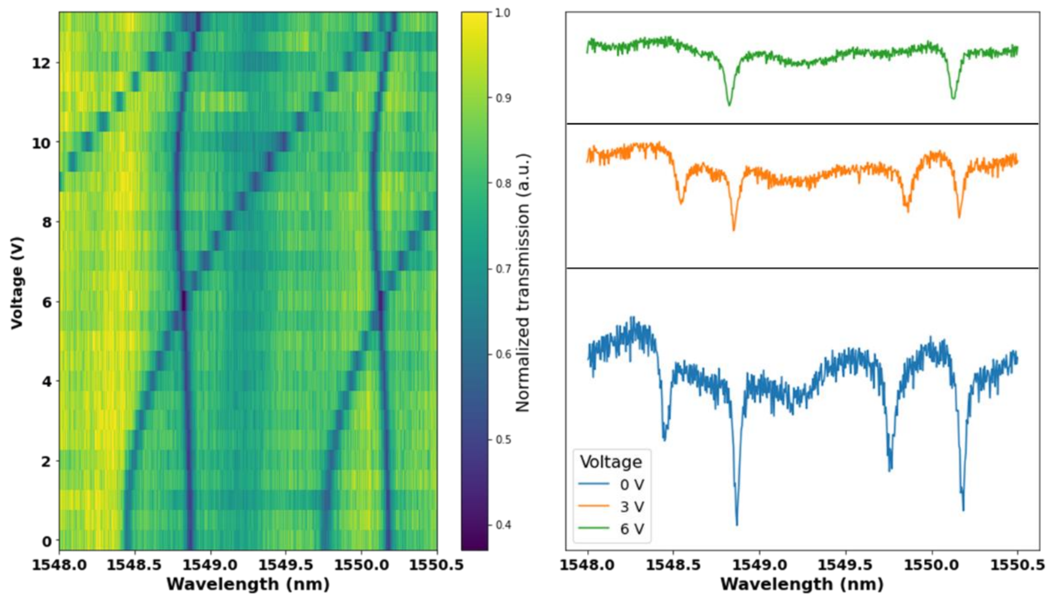

Figure S18. *Transmission intensity as function of the wavelength spectra for different applied voltages for the device in fig.3b-c of the main manuscript and wavelength spectra for voltages 0V, 3V and 6V taken from the 2D map. The spectra are separate between each other.*

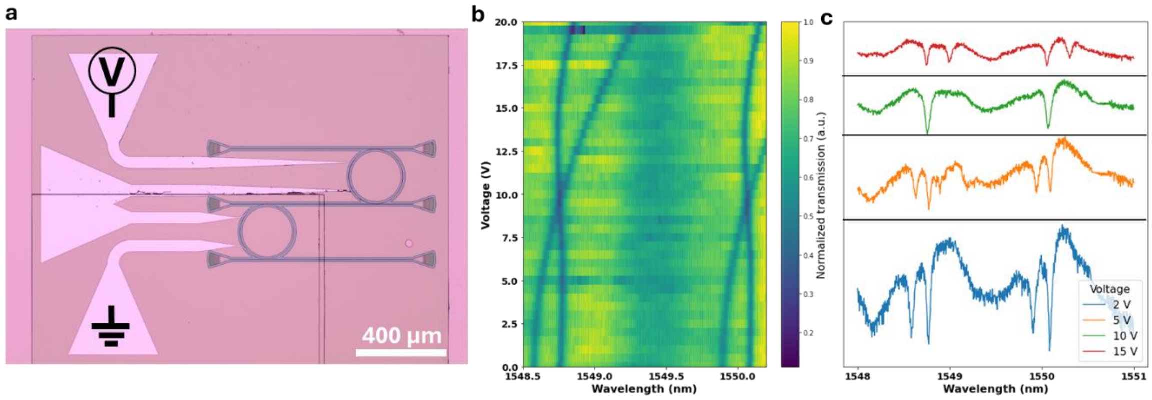

Figure S19. a) *Optical microscope image of device 2 (shown in the main article fig.3b-c) connected in series with two ring resonators with a middle waveguide to track the resonance dips.* ***b)*** *Wavelength intensity spectra as a function of the volage applied and* ***c)*** *Wavelength spectra for 2V (blue), 5V (orange), 10V (green) and 15V (red). The spectra are separate between each other.*

7. Thermal crosstalk

In this section we show the corresponding spectra taken at different voltages (from 0V to 10V) to characterize the thermal crosstalk between devices using continuous PECVD (**fig.S20**) and ICPCVD (**fig.S21**) claddings as well as cladding deposited using lift-off for thermal isolation (**fig.S22**). Ring A depicts the device where the heater is applied while Ring B is the device that shifts due to thermal crosstalk. We also show an SEM image of the region between optical devices after performing ICPCVD lift-off of the cladding (**fig.S23**).

7.1. PECVD Continuous films

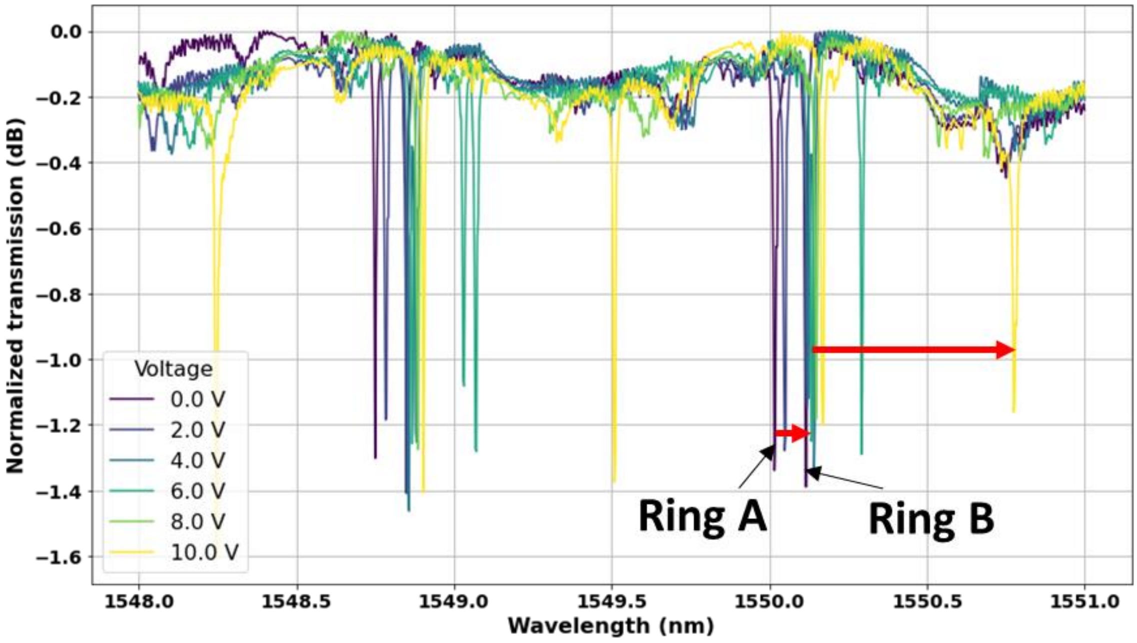

Figure S20. *Spectra at different voltages for the two ring resonators fabricated using continuous PECVD cladding*

7.2. ICPCVD Continuous film

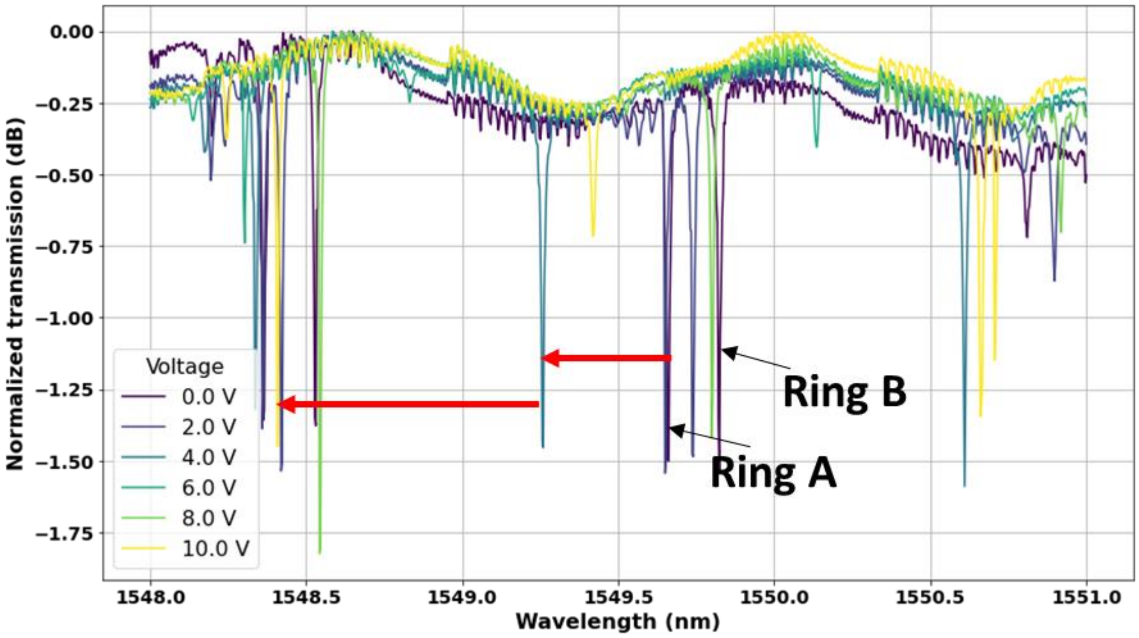

Figure S21. *Spectra at different voltages for the two ring resonators fabricated using continuous ICPCVD cladding.*

7.3. ICPCVD lift-off cladding

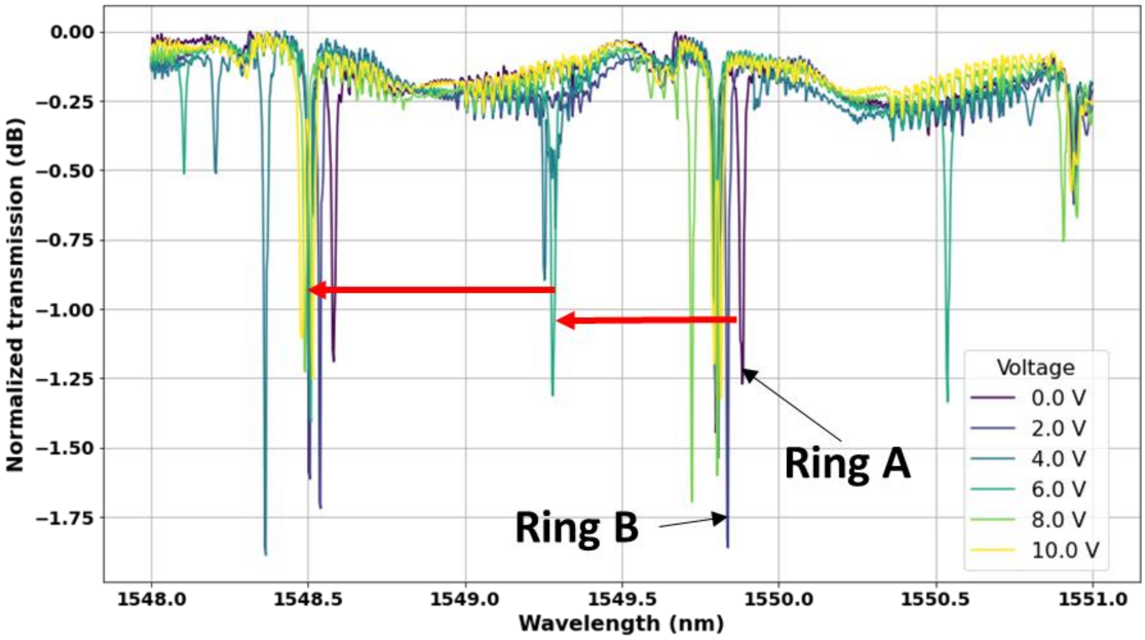

Figure S22. *Spectra at different voltages for the two ring resonators fabricated using the lift-off approach.*

We performed scanning electron microscopy (SEM) of the cladding fabricated using lift-off and the resulting image is shown in fig.S23. Depositing a cladding delimited by resist produces roughness in the region where the lift-off is performed. The main reason is that the silicon dioxide is also deposited with lower quality without continuity on the resist. When the lift-off is performed, material in the cladding that is also attached to the resist is also removed. It is important to notice that this does not affect the optical quality of the mode since the cladding is wide enough (cladding width of 9 µm) . In the picture below is also pointed the top micro-heater width (yellow), the remaining amorphous silicon carbide film (orange), the limit of the silicon dioxide cladding (blue) and the bottom thermal silicon dioxide (red).

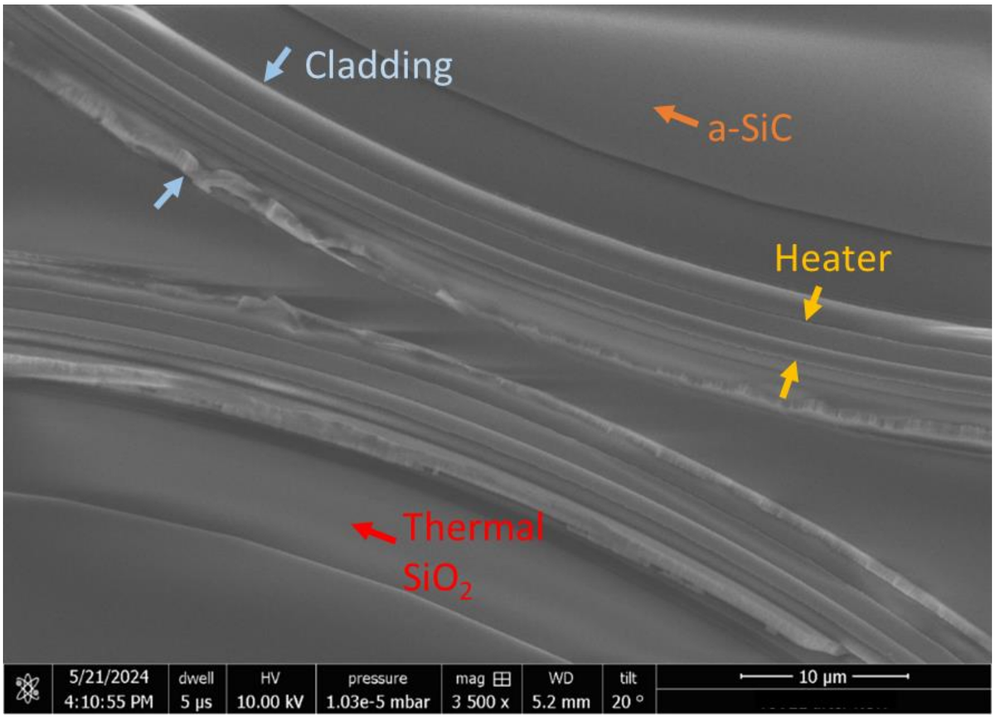

Figure S23. *Scanning Electron Microscope imaging of the resulting cladding after lift-off in the region between two optical ring resonators with the different elements delimited with arrows: cladding (blue), heaters, (yellow), a-SiC film (orange) and thermal silicon dioxide (red).*

8. Ramp-up and ramp-down measurements

To address the repeatability of the measurements using the different claddings, we performed forward and backward voltage sweeps on devices with ICPCVD and PECVD silicon dioxide. The voltage was varied between 0V and 6V. **Fig.S24a** summarize the data for two different devices with similar ring resonator parameters as stated in the main manuscript and using ICPCVD cladding deposited at a temperature of 150°C and chamber pressure of 8 mTorr. Device 1 shows a deviation between measurements that could be related to device fabrication but also hysteresis. The second device presents a smaller variation and expected behaviour. **Fig.S24b** shows the wavelength shift as a function of the voltage for a device with PECVD cladding deposited at 300°C compared to the data in **fig.S24a**. In comparison, the measurement results using a PECVD silicon dioxide cladding show a smaller deviation between data points and no signs of hysteresis.

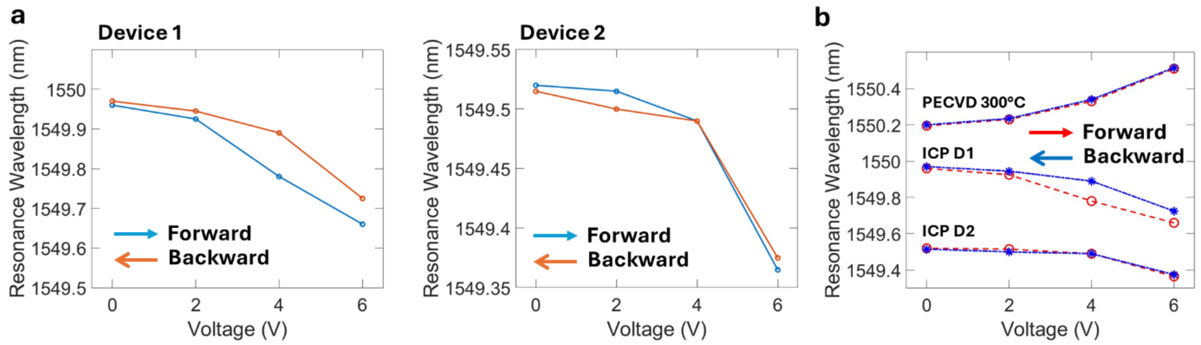

Figure S24. *Forward and backward voltage sweeps of a-SiC ring resonators made with silicon dioxide deposited with **a)** ICPCVD at 150°C and chamber pressure of 8 mTorr for two different devices and **b)** comparison with a cladding deposited with PECVD at 300°C.*

9. Stability measurements of the optical setup

To assess the reproducibility of the measurements and determine the error given by the optical setup with the tunable laser, we performed four consecutive sweeps of temperature between 27°C and 31°C on a sample with ICPCVD silicon dioxide cladding deposited at 150°C and a chamber pressure of 10 mTorr. We extracted the thermal shifts with the respective errors in the fitting and determined the standard deviation of the measured resonant wavelength at a fixed stage temperature as summarized in **table S12.**

	λ at 27°C (nm)	λ at 29°C (nm)	λ at 31°C (nm)	Slope (pm/°C)
**Ramp 1**	1549.271	1549.142	1548.887	-96 ± 18
**Ramp 2**	1549.276	1549.085	1548.887	-97 ± 1
**Ramp 3**	1549.235	1549.109	1548.902	-83 ± 12
**Ramp 4**	1549.319	1549.157	1548.935	-96 ± 9
**Standard deviation**	0.034	0.032	0.023	

Table S12. *Resonance wavelength position as a function of temperature for the four consecutive measurements together with the extracted slope and error.*
